# Association of Neighborhood Disadvantage With Cardiovascular Risk Factors and Events Among Refugees in Denmark

**DOI:** 10.1001/jamanetworkopen.2020.14196

**Published:** 2020-08-21

**Authors:** Rita Hamad, Buket Öztürk, Else Foverskov, Lars Pedersen, Henrik T. Sørensen, Hans E. Bøtker, Justin S. White

**Affiliations:** 1 Philip R. Lee Institute for Health Policy Studies, University of California School of Medicine, San Francisco; 2 Department of Family & Community Medicine, University of California School of Medicine, San Francisco; 3 Department of Clinical Epidemiology, Aarhus University, Denmark; 4 Center for Population Health Science, Stanford University, Stanford, California; 5 Department of Cardiology, Aarhus University Hospital, Denmark; 6 Department of Epidemiology & Biostatistics, University of California School of Medicine, San Francisco

## Abstract

**Importance:**

Refugees are among the most disadvantaged individuals in society, and they often have elevated risks of cardiovascular risk factors and events. Evidence is limited regarding factors that may worsen cardiovascular health among this vulnerable group.

**Objective:**

To test the hypothesis that refugee placement in socioeconomically disadvantaged neighborhoods is associated with increased cardiovascular risk.

**Design, Setting, and Participants:**

The study population of this quasi-experimental, registry-based cohort study included 49 305 adults 18 years and older who came to Denmark as refugees from other countries during the years of Denmark’s refugee dispersal policy from 1986 to 1998. Refugees were dispersed to neighborhoods with varying degrees of socioeconomic disadvantage in an arbitrary manner conditional on observed characteristics. The association of neighborhood disadvantage on arrival with several cardiovascular outcomes in subsequent decades was evaluated using regression models that adjusted for individual, family, and municipal characteristics. Health outcomes were abstracted from the inpatient register, outpatient specialty clinic register, and prescription drug register through 2016. Data analysis was conducted from May 2018 to July 2019.

**Exposures:**

A composite index of neighborhood disadvantage was constructed using 8 neighborhood-level socioeconomic characteristics derived from Danish population register data.

**Main Outcomes and Measures:**

Primary study outcomes included hypertension, hyperlipidemia, type 2 diabetes, myocardial infarction, and stroke. Before data analysis commenced, it was hypothesized that higher levels of neighborhood disadvantage were associated with an increased risk of cardiovascular risk factors and events.

**Results:**

A total of 49 305 participants were included (median [interquartile range] age, 30.5 [24.9-39.8] years; 43.3% women). Participant region of origin included 6318 from Africa (12.8%), 7253 from Asia (14.7%), 3446 from Eastern Europe (7.0%), 5416 from Iraq (11.0%), 6206 from Iran (12.6%), 5558 from Palestine (via Lebanon, Israel, Occupied Palestinian Territories; 11.3%), and 15 108 from Yugoslavia (30.6%). Adjusted models revealed an association between placement in disadvantaged neighborhoods and increased risk of hypertension (0.71 [95% CI, 0.30-1.13] percentage points per unit of disadvantage index; *P* < .01), hyperlipidemia (0.44 [95% CI, 0.06-0.83] percentage points; *P* = .01), diabetes (0.45 [95% CI, 0.09-0.81] percentage points; *P* = .01), and myocardial infarction (0.14 [95% CI, 0.03-0.25] percentage points; *P* = .01). No association was found for stroke. Individuals who arrived in Denmark before age 35 years had an increased risk of hyperlipidemia (1.16 [95% CI, 0.41-1.92] percentage points; *P* < .01), and there were no differences by sex.

**Conclusions and Relevance:**

In this quasi-experimental cohort study, neighborhood disadvantage was associated with increased cardiovascular risk in a relatively young population of refugees. Neighborhood characteristics may be an important consideration when refugees are placed by resettlement agencies and host countries. Future work should examine additional health outcomes as well as potential mediating pathways to target future interventions (eg, neighborhood ease of walking, employment opportunities).

## Key Points

**Question:**

Is there an association of neighborhood socioeconomic disadvantage with the development of cardiovascular risk factors, myocardial infarction, and stroke among refugees?

**Findings:**

In this quasi-experimental cohort study, 49 305 refugees who were assigned to more disadvantaged neighborhoods across Denmark were at increased risk of developing hypertension, hyperlipidemia, diabetes, and myocardial infarction over subsequent decades. No associations were found for stroke.

**Meaning:**

Neighborhood characteristics may be associated with long-term cardiovascular risk among refugees.

## Teaser

This quasi-experimental cohort study uses data from the Danish population register of adult immigrants arriving from 1986 to 1998 and health outcomes from the inpatient clinic register, outpatient clinic register, and prescription drug register to assess the association between disadvantaged neighborhoods and cardiovascular disease risk among refugees.

## Introduction

Refugees are among the most vulnerable individuals in society, often surviving violence, war, and other trauma before relocating to an unfamiliar host country.^1,2^ Although some immigrants have better health than native-born citizens,^3^ refugees are uniquely at risk due to the often involuntary and sudden nature of their displacement and the adversity faced in their home countries.^4,5^ In many countries, population-level studies of refugee health are notoriously difficult to conduct due to data constraints. For example, the United States collects minimal data on refugees only during the 90 days after their arrival.^6^ Most literature is therefore limited to a few small cross-sectional studies of refugees from a single country or region or to the few refugees who happen to be interviewed in national surveys.^5,7,8,9^

A few studies have demonstrated that rates of cardiovascular disease (CVD) and related risk factors among some refugees are increased compared with those of native-born populations.^10,11,12,13,14^ In the general population, CVD is more common among individuals of lower socioeconomic status and is associated with chronic stress,^15,16,17^ making it a particularly relevant outcome among refugees.

Previous studies have investigated factors associated with health among refugees, although most focused on mental health as an outcome and on individual risk factors, such as sex or exposure to violence.^18^ Fewer studies focused on neighborhood factors,^18^ which theory suggests are key determinants of immigrant health.^19^ For example, socioeconomically disadvantaged neighborhoods often have limited walkability or availability of nutritious food, thereby affecting health behaviors associated with CVD.^20,21,22^ Alternatively, reduced employment opportunities or greater neighborhood crime rates may increase stress, which in turn is associated with increased CVD.^23,24,25,26,27^ Neighborhood socioeconomic characteristics may be particularly salient for refugees, who often have limited control over their placement and limited financial means.

This study took advantage of a unique natural experiment in which refugees to Denmark were assigned to neighborhoods with different levels of socioeconomic disadvantage.^28^ From 1986 to 1998, the Danish government dispersed tens of thousands of incoming refugees across the country in an arbitrary fashion, conditional on observed characteristics, to avoid overcrowding in major cities.^27,28,29,30,31^ We employed unique population-level data spanning 3 decades from Denmark’s population and health registers. We tested the hypothesis that neighborhood socioeconomic disadvantage is associated with increased risk of CVD risk factors, myocardial infarction, and stroke across the life course among resettled refugees.

## Methods

### Ethics Approval

This quasi-experimental, registry-based cohort study was approved by the Danish Data Protection Agency (record number 2015-57). Registry-based studies do not require ethical board approval in Denmark,^32^ and this study did not include any personally identifiable information on study participants. As a result, participant consent was not obtained.

### Data

The cohort was created by linking several Danish national registers.^33^ Sociodemographic data were drawn from administrative registers of the total population (January 1, 1986, to December 31, 2016), whereas outcomes were ascertained from inpatient (January 1, 1986, to December 31, 2016), outpatient specialty clinic (January 1, 1994, to December 31, 2016), and prescription drug (January 1, 1995, to December 31, 2016) registers.^34,35,36^ Danish registers do not include diagnostic information on primary care encounters. The registers include 8.1 million individuals cumulatively during the study period. Data analysis was conducted from May 2018 to July 2019. This study followed the Strengthening the Reporting of Observational Studies in Epidemiology (STROBE) reporting guideline.

### Study Population

The term *immigrants* refers to foreign-born individuals, whereas *refugees* are a unique subset of immigrants fleeing persecution and entitled to specific protections under international law. The study population included adult immigrants (18 years and older) arriving in Denmark from refugee-sending countries during the 1986 to 1998 period, which represents the years of the government dispersal policy (N = 49 305; eFigure 1 in the Supplement). We excluded individuals reuniting with family in Denmark, because they were not subject to the dispersal policy.

### Danish Dispersal Policy

**Figure 1.**
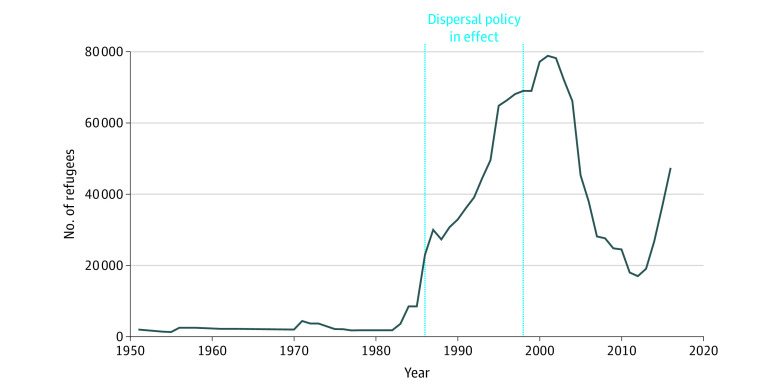
Refugees to Denmark by Year Source: Authors’ calculations using data from the United Nations High Commissioner for Refugees (http://popstats.unhcr.org).

**Figure 2.**
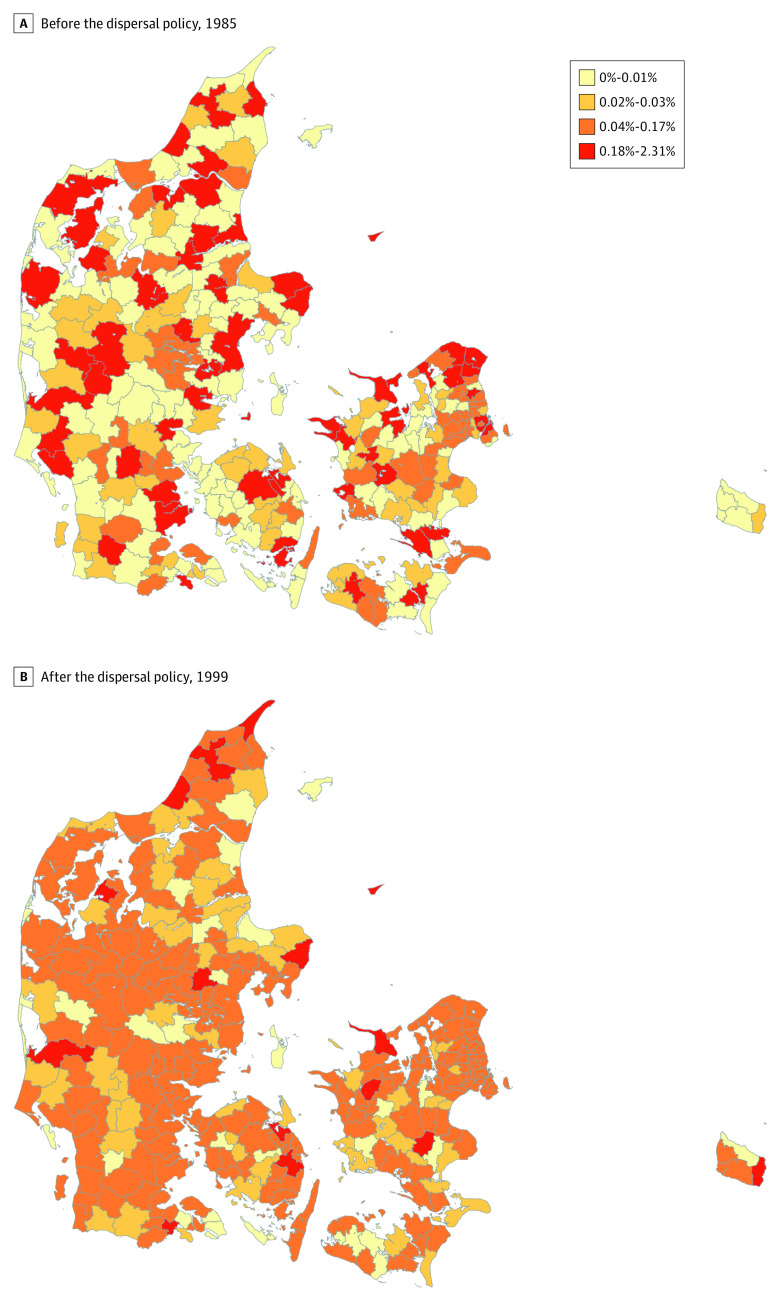
Percentage of Immigrants From Countries Sending Refugees to Denmark by Municipality Percentage of immigrants shown in the year before (A) and the year after (B) the dispersal policy was in effect (1985 vs 1999). Source: Authors’ calculations using data from Statistics Denmark.

We leveraged a unique natural experiment in which incoming refugees were assigned to neighborhoods with varying levels of disadvantage throughout the country. Denmark implemented this refugee dispersal policy from 1986 to 1998.^28^ Because of the surge in arrivals to Denmark during this period (Figure 1), the policy’s goal was to ease labor market conditions in heavily settled areas and to promote better integration of refugees. As a result of the policy, refugees were spread more evenly across the country (Figure 2).

Refugee placement officers only had knowledge of the following characteristics that were available on refugees’ applications: sex, birthdate, marital status, family size, and nationality.^28^ Officers otherwise had no direct interaction with families. Therefore, any possible confounding would have arisen only from observed characteristics in the refugees' applications, reducing the chances of confounding by unmeasured factors (ie, guaranteeing conditional exchangeability).^37,38^ Thus, initial neighborhood assignment within a municipality was arbitrary, conditional on these demographic characteristics. These characteristics are available in the register data, and we adjusted for them during data analysis. Studies have identified greater geographic dispersal of refugees during the policy period (as in Figure 2), no evidence of sorting by ethnic group, and covariate balance by neighborhood type.^27,28,29,30^ It is therefore unlikely that unobserved factors influenced neighborhood assignment. As a result, this study attempts to address the challenges of selective migration present in existing studies on neighborhood outcomes.

Although register data do not include information on the neighborhood to which refugees were assigned, official reports document that more than 90% of incoming refugees agreed to placement in their assigned municipality, indicating strong adherence to the placement program.^28^ Those who refused placement were responsible for finding their own place of residence, which may have resulted in some residual bias in our estimates. Nevertheless, this represents a high level of compliance relative to the only prior relevant assignment study to our knowledge, the US Moving to Opportunity experiment, in which only half of participants adhered to their assignment.^39^ As refugees reuniting with family were not subject to the dispersal policy, we excluded these individuals using family structure information available in the registers. For the remaining refugees, we assumed that the observed neighborhood of residence on arrival was the neighborhood to which refugees were assigned. For those who moved within 1 year of arrival, we assumed that the first place of residence was temporary housing—similar to prior work based on how the policy was implemented^28,29^—and instead used the second place of residence as the assigned neighborhood.

After assignment to an initial residence, the government offered language and training courses and social welfare support lasting approximately 18 months. There were no relocation restrictions if a family later decided to move, and receipt of welfare was not conditional on remaining in the assigned residence. Therefore, our study design is akin to a randomized encouragement design in which participants are randomly encouraged to be exposed to a given neighborhood.^40^ The approach provides a lower bound on the health outcomes associated with neighborhood disadvantage. The design is similar to that used for the Moving to Opportunity experiment.^39^ Later relocations represent mediating pathways through which disadvantage in the initially assigned neighborhood may be associated with later cardiovascular health.

### Variables

#### Exposure

For each individual, the primary exposure was a measure of socioeconomic disadvantage in the initial neighborhood assigned on arrival to Denmark. We created a composite disadvantage index for each neighborhood by year, using principal component analysis to combine 8 neighborhood-level sociodemographic variables examined in prior research that represent different theoretical constructs capturing disadvantage: median income, family poverty, income inequality, unemployment rate, crime rate, foreign-born, welfare participation, and education.^41,42^ Additional details are available in the eAppendix and eTables 1 and 2 in the Supplement.

Small geographic units were used to define neighborhoods, as these align better than larger areas with residents’ definitions of neighborhoods.^43^ Using larger areas can also mask neighborhood effects.^44^ The Danish census bureau has geocoded all individuals to historically meaningful neighborhoods known as parishes. Parishes are nested within the larger geographic unit of municipality and each contain a mean of approximately 2000 individuals. During the study period, there were 2159 parishes nested within 271 municipalities, and they have been used previously to define neighborhoods.^45,46^

#### Outcomes

We examined hypertension, hyperlipidemia, type 2 diabetes, myocardial infarction (MI), and stroke, which represent 3 CVD risk factors and 2 clinical end points that have been plausibly linked to neighborhood disadvantage previously.^47,48,49,50,51,52,53,54^ For MI and stroke, individuals were considered to have these diagnoses if they were assigned relevant *International Classification of Diseases, Eighth Revision (ICD-8)* and *International Statistical Classification of Diseases and Related Health Problems, Tenth Revision (ICD-10)* codes, based on physician discharge diagnoses in inpatient settings. For hypertension, hyperlipidemia, and diabetes, we used *ICD-8* and *ICD-10* codes from outpatient specialty clinics and inpatient settings, as well as Anatomic Therapeutic Chemical medication codes from prescriptions of relevant medications (eg, statins for hyperlipidemia). To capture incident rather than prevalent cases, we excluded diagnoses within 2 years after arriving in Denmark.^55,56^ We also extracted the date on which diagnoses were first identified, which we used in Cox models.

#### Covariates

We adjusted for the characteristics available to placement officers who implemented the dispersal policy: sex, age and age-squared, marital status, family size, and region of origin. We included indicator variables for year of arrival to adjust for secular trends. Finally, we included indicator variables (ie, fixed effects) for municipality, which accounted for all time-invariant characteristics of municipalities. This allowed us to estimate associations with neighborhood disadvantage based on the difference in outcomes for refugees placed in the same municipality who were assigned to a high- vs low-disadvantage neighborhood.

### Statistical Analysis

#### Primary Analysis

We first examined characteristics of individuals in the study population, splitting the neighborhood disadvantage index at the median (for the purposes of this descriptive analysis only) and comparing individuals assigned to high- vs low-disadvantage neighborhoods. We also compared the prevalence of each outcome among the refugee population with the prevalence among an age- and sex-matched population of native-born Danes.

Next, we regressed each outcome on the continuous measure of neighborhood disadvantage. In primary analyses, these were linear regressions, and we confirmed a linear association between the disadvantage index and each outcome through a graphical spline analysis. The models were first unadjusted and then adjusted for the covariates listed previously. All tests were 2-sided, and *P* < .05 was considered statistically significant; 95% CIs indicated precision of effect estimates. Robust SEs were clustered by municipality to account for correlated observations.

#### Secondary Analyses

Linear models do not consider the date of diagnosis and therefore do not capture the time to event, which may be important for these outcomes. To account for this possible bias, we next used Cox models to estimate the association of neighborhood disadvantage with time-to-diagnosis for each outcome. These models were first unadjusted and then adjusted for the covariates mentioned previously. They incorporated shared frailty at the municipality level to account for correlated observations. See the eAppendix in the Supplement for additional details.

Finally, we assessed whether associations differed by sex and by age on arrival, as men and women may experience neighborhood disadvantage differently, and individuals exposed at younger ages experience longer exposure to a neighborhood after arrival.^57^ We carried out an analysis in which we included an interaction term between neighborhood disadvantage and sex and another with an interaction term between neighborhood disadvantage and age younger than 35 years on arrival.

## Results

### Cohort Characteristics

A total of 49 305 participants were analyzed (median [interquartile range] age, 30.5 [24.9-39.8] years; 43.3% women) (Table). More than half (28 629 [58.1%]) were married, and approximately one-fifth had completed secondary education (14.4%) or more (5.8%). Participant region of origin included 6318 from Africa (12.8%), 7253 from Asia (14.7%), 3446 from Eastern Europe (7.0%), 5416 from Iraq (11.0%), 6206 from Iran (12.6%), 5558 from Palestine (via Lebanon, Israel, Occupied Palestinian Territories; 11.3%), and 15 108 from Yugoslavia (30.6%). Characteristics were roughly balanced across high- and low-disadvantage neighborhoods, except for the characteristics that were available to placement officers and may have influenced neighborhood assignment (eg, region of origin) and those correlated with such characteristics (eg, educational attainment). The median follow-up time was 16.0 (interquartile range, 3.7-20.3) years.

**Table.** Sociodemographic characteristics of Refugees Arriving in Denmark During 1986-1998, by Neighborhood Disadvantage Level on Arrival

Characteristic^a^	Low-disadvantage neighborhood (n = 24 656)		High-disadvantage neighborhood (n = 24 649)		Total (N = 49 305)	
	%	Median (IQR)	%	Median (IQR)	%	Median (IQR)
Women	42.3	NA	44.3	NA	43.3	NA
Age, y	NA	29.9 (24.6-38.8)	NA	31.3 (25.2-40.7)	NA	30.5 (24.9-39.8)
Married	54.9	NA	61.2	NA	58.1	NA
Educational level						
No education	26.8		24.8		25.8	
Primary education	51.5		56.4		53.9	
Secondary education	15.2		13.6		14.4	
Higher education	6.4		5.2		5.8	
Family size, No. of people	NA	2 (1-3)	NA	2 (1-4)	NA	2 (1-4)
Region of origin						
Africa	13.9	NA	11.7	NA	12.8	NA
Asia	12.8		16.6		14.7	
Eastern Europe	8.5		5.5		7.0	
Iraq	13.9		8.1		11.0	
Iran	15.0		10.2		12.6	
Palestine^b^	13.7		8.8		11.3	
Yugoslavia	22.1		39.2		30.6	
Follow-up, y	NA	14.5 (3.3-21.1)	NA	17.1 (4.1-20.1)	NA	16.0 (3.7-20.3)
Health outcomes						
Hypertension	36.5	NA	37.8	NA	37.2	NA
Hyperlipidemia	23.3		27.1		25.2	
Diabetes	15.6		16.4		16.0	
Myocardial infarction	3.2		3.4		3.3	
Stroke	2.7		2.8		2.8	

Abbreviations: IQR, indicates interquartile range; NA, not applicable.

^a^ Study population includes all adult immigrants (aged 18 years and older) who arrived in Denmark from countries sending refugees during the period 1986-1998. Diagnoses were extracted from register data using medication and physician diagnosis codes. A composite index of socioeconomic disadvantage was created for each neighborhood by year, using principal component analysis to combine 8 neighborhood-level sociodemographic variables (eTable 1 in the Supplement). For the purposes of producing these descriptive characteristics only, the neighborhood disadvantage index was split at the median to compare refugees assigned to low- vs high-disadvantage neighborhoods.

^b^ The designation *Palestine* includes individuals arriving from Lebanon, Israel, or the Occupied Palestinian Territories.

During the follow-up period, 37.2% of refugees were diagnosed with hypertension, 25.2% with hyperlipidemia, 16.0% with diabetes, 3.3% with MI, and 2.8% with stroke. Rates of all conditions except stroke were higher among the refugee population compared with a population of native-born Danes matched by age and sex (hypertension, 37.2% vs 35.3%; hyperlipidemia, 25.2% vs 18.7%; diabetes, 16.0% vs 7.1%; MI, 3.3% vs 2.7%; stroke 2.8% vs 3.6%) (eTable 3 in the Supplement).

### Association of Neighborhood Disadvantage With Cardiovascular Risk Factors and Events

**Figure 3.**
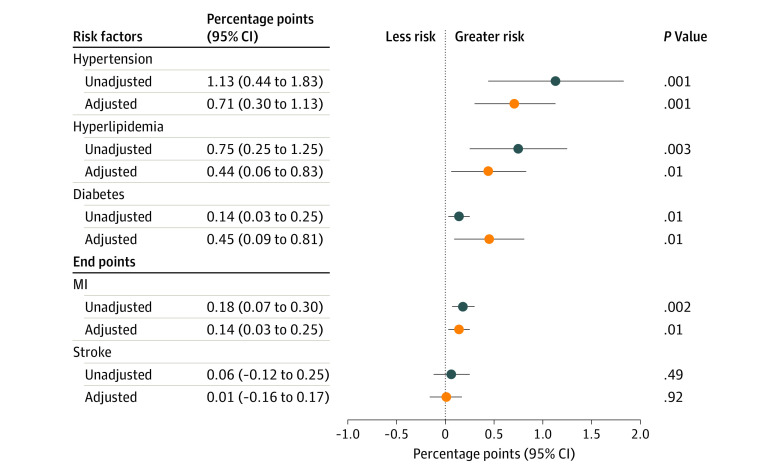
Association of Neighborhood Disadvantage With Cardiovascular Outcomes, N = 49 305 The study population includes all adult immigrants (aged 18 years and older) who arrived in Denmark from countries sending refugees from 1986 to 1998. Diagnoses were extracted from register data using medication and physician diagnosis codes. A continuous variable representing a composite index of socioeconomic disadvantage was created for each neighborhood by year using principal component analysis to combine 8 neighborhood-level sociodemographic variables (eTable 1 in the Supplement). Analyses involved multivariable linear regressions, with covariates including family size, sex, marital status, region of origin, year of arrival, and fixed effects for municipality. Coefficients represent the change in risk (in percentage points) per unit of the disadvantage index, with 95% CIs in parentheses for both risk factors and end points. MI indicates myocardial infarction.

In unadjusted models (Figure 3), greater neighborhood disadvantage was associated with a greater risk of hypertension (1.13 [95% CI, 0.44-1.83] percentage points per unit of deprivation index; *P* = .001), hyperlipidemia (0.75 [95% CI, 0.25-1.25] percentage points; *P* = .003), diabetes (0.14 [95% CI, 0.03-0.25] percentage points; *P* = .01), and MI (0.18 [95% CI, 0.07-0.30] percentage points; *P* = .002) but had no association with stroke.

In adjusted models, greater neighborhood disadvantage was associated with increased risk of hypertension (0.71 [95% CI, 0.30-1.13] percentage points; *P* < .01), hyperlipidemia (0.44 [95% CI, 0.06-0.83] percentage points; *P* = .01), diabetes (0.45 [95% CI, 0.09-0.81] percentage points; *P* = .01), and MI (0.14 [95% CI, 0.03-0.25] percentage points; *P* = .01), but not with stroke.

In Cox models, greater neighborhood disadvantage was associated with increased risk of diabetes (adjusted hazard ratio, 1.03; 95% CI, 1.01-1.06; *P* = .04) (eFigure 2 in the Supplement). However, we were unable to rule out the null hypothesis of no association for other outcomes.

### Subgroup Analyses

Differences in the association between neighborhood disadvantage and each outcome were similar for men and women (eTable 4 in the Supplement). Increased neighborhood disadvantage was associated with a greater likelihood of hyperlipidemia among refugees who arrived before age 35 years compared with those who arrived at older ages (1.16 [95% CI, 0.41-1.92] percentage points; *P* < .01) (eTable 4 in the Supplement). For other outcomes, there were no differences in the association of neighborhood disadvantage among those who arrived in Denmark at younger or older ages.

## Discussion

This quasi-experimental cohort study leveraged a unique natural experiment and rich Danish register data to provide among the first rigorous estimates of the association of neighborhood disadvantage with cardiovascular risk factors, MI, and stroke among refugees. Study results suggest that refugees who were assigned to more disadvantaged neighborhoods were more likely to develop hypertension, hyperlipidemia, diabetes, and MI in subsequent decades. Effect sizes were small, representing a 2% increase from baseline rates for each condition; although not necessarily clinically meaningful, these rates signify more meaningful outcomes when considering changes in distribution at a population level.^58^ We found no association of neighborhood disadvantage with risk of stroke, although results suggest that younger individuals were more likely than older individuals to develop hyperlipidemia in association with living in a more disadvantaged neighborhood.

Our findings are consistent with prior evidence from numerous observational studies that suggest that neighborhood characteristics are associated with CVD and its risk factors.^47,48,49,50,51,59,60,61,62,63^ Although many previous studies were limited by potential reverse association or confounding due to possible selection of unhealthy individuals (ie, those with higher CVD risk at baseline) into more disadvantaged neighborhoods, our use of a natural experiment reduced these sources of bias. One prior study that took advantage of a similar natural experiment in Sweden found that assignment to a more disadvantaged neighborhood was associated with higher rates of diabetes among refugees in later decades of life.^53^ This finding strengthens the argument that neighborhood socioeconomic circumstances matter for CVD risk.

Numerous mechanisms may underlie our findings. For example, disadvantaged neighborhoods may constrain employment and economic opportunities, thereby reducing income and the ability to purchase nutritious food.^61^ In addition, more disadvantaged neighborhoods often have more restricted food environments and walkability, thereby affecting health behaviors related to CVD.^20,21,22^ These neighborhoods also may have poorer access to primary care and other health care resources, resulting in lower-quality prevention and treatment of health conditions. Notably, health care access in Denmark is more equitably available nationwide relative to the United States.^64,65,66^ In addition, reduced income and greater neighborhood crime may increase chronic stress levels, which have been associated with increased rates of CVD.^23,24,25,26^ However, 1 prior study found that neighborhood disadvantage was not associated with refugee mental health in the context of a similar refugee dispersal policy in Sweden.^67^

Our results also suggest that, for hyperlipidemia, these pathways are more salient for refugees who arrive at younger ages. This is consistent with the literature on sensitive periods earlier in life that might constitute windows in which social exposures are particularly important.^57,68^ Future studies are needed to identify additional individual- and neighborhood-level characteristics that may place individuals at greater risk.

Previous work also has demonstrated that neighborhood disadvantage is associated with an increased risk of stroke,^48,69,70,71^ which we did not confirm in this study. Although it is possible that prior studies suffered from confounding and that there is, in fact, no effect of neighborhood disadvantage on this outcome, our study population may have been too young and this outcome too uncommon to replicate previous findings.

### Limitations

Our study has several limitations. First, the study population included only refugees to Denmark, so the results may not generalize to refugees in other settings or to nonrefugees. Also, our study is unique due to the presence of the dispersal policy that created a natural experiment, and the outcomes of neighborhood disadvantage may differ when individuals self-select their place of residence. In addition, the dispersal policy that we examined was implemented during the 1986 to 1998 period, and the role of neighborhood disadvantage may differ based on other contemporaneous factors, such as economic conditions. Future studies should seek to identify more recent natural experiments. Additionally, the Danish register data include information on neighborhood of residence rather than neighborhood assigned. Although official reports document adherence of over 90% to the dispersal policy, there may nevertheless be some bias in resulting estimates. One limitation of Cox models is possible differential left-truncation bias, as various registers were established at different times. If differences in start date are associated with levels of neighborhood disadvantage and dates of diagnosis, Cox models could be biased. Finally, our study does not elucidate the specific socioeconomic aspects of neighborhoods that may underlie our findings, because we used a composite measure of neighborhood disadvantage. Future studies could examine how different aspects of neighborhoods—eg, unemployment, crime, walkability—may interact in order to develop targeted community interventions.

## Conclusions

Given the surge in refugee migration to high-income countries in recent years,^72^ evidence is needed to inform governmental policies to optimize the economic and health trajectories of this vulnerable group. Such evidence can inform the development of tools to guide placement of refugees by host countries. Available tools currently do not include neighborhood-level characteristics.^73^

The findings of this quasi-experimental, registry-based cohort study suggest that neighborhoods where refugees are placed may have an association with risk of several CVD outcomes. Future studies should examine whether results are stronger after additional years of follow-up and aging of the cohort and should include additional outcomes. Additional studies could assess whether other subgroups (eg, those defined by region of origin or psychological resiliency) are particularly susceptible to neighborhood circumstances.

## Notes

Supplement. **eAppendix.** Supplemental Methods **eFigure 1.** Study Flowchart **eFigure 2.** Effect of Neighborhood Disadvantage on Cardiovascular Outcomes, Cox Models **eFigure 3.** Effect of Neighborhood Disadvantage on Cardiovascular Outcomes, Using Alternative Neighborhood Deprivation Index **eTable 1.** Characteristics Included in Neighborhood Disadvantage Index **eTable 2.** Principal Component Analysis: Eigenvalues and Proportion of Variance Explained **eTable 3.** Prevalence of Outcomes in Refugee Population Compared With Population of Native-Born Danes Matched on Age and Sex **eTable 4.** Effect of Neighborhood Disadvantage on Cardiovascular Outcomes, by Sex and Age of Arrival in Denmark **eReferences**
