# Supplementary material for: Association of Neighborhood Disadvantage With Cardiovascular Risk Factors and Events Among Refugees in Denmark
Source: JAMA Netw Open. 2020 Aug 21;3(8):e2014196. doi: 10.1001/jamanetworkopen.2020.14196 (PMC7442927; doi:10.1001/jamanetworkopen.2020.14196)
Supplement: Supplement. — eAppendix. Supplemental Methods eFigure 1. Study Flowchart eFigure 2. Effect of Neighborhood Disadvantage on Cardiovascular Outcomes, Cox Models eFigure 3. Effect of Neighborhood Disadvantage on Cardiovascular Outcomes, Using Alternative Neighborhood Deprivation Index eTable 1. Characteristics Included in Neighborhood Disadvantage Index eTable 2. Principal Component Analysis: Eigenvalues and Proportion of Variance Explained eTable 3. Prevalence of Outcomes in Refugee Population Compared With Population of Native-Born Danes Matched on Age and Sex eTable 4. Effect of Neighborhood Disadvantage on Cardiovascular Outcomes, by Sex and Age of Arrival in Denmark eReferences [file jamanetwopen-3-e2014196-s001.pdf]

Supplementary Online Content

Hamad R, Öztürk B, Foverskov E, et al. Association of neighborhood disadvantage with cardiovascular risk factors and events among refugees in Denmark. *JAMA Netw Open*. 2020;3(8):e2014196. doi:10.1001/jamanetworkopen.2020.14196

eAppendix. Supplemental Methods

eFigure 1. Study Flowchart

eFigure 2. Effect of Neighborhood Deprivation on Cardiovascular Outcomes, Cox Models

eFigure 3. Effect of Neighborhood Deprivation on Cardiovascular Outcomes, Using Alternative Neighborhood Deprivation Index

eTable 1. Characteristics Included in Neighborhood Deprivation Index

eTable 2. Principal Component Analysis: Eigenvalues and Proportion of Variance Explained

eTable 3. Prevalence of Outcomes in Refugee Population Compared With Population of Native-Born Danes Matched on Age and Gender

eTable 4. Effect of Neighborhood Deprivation on Cardiovascular Outcomes, by Gender and Age of Arrival in Denmark

eReferences

This supplementary material has been provided by the authors to give readers additional information about their work.

© 2020 Hamad R et al. *JAMA Network Open.*

eAppendix. Supplemental Methods

Register Data

Validation studies have found Denmark's clinical registers to be of high quality and virtually 100% complete.[1-5] Abstracting diagnostic information from clinical encounter data is an established technique; the ascertainment scheme used in this study has been systematically and repeatedly validated in the Danish registers against clinical diagnostic algorithms, with high sensitivity and specificity.[6-10] Since virtually all healthcare utilization is captured in the register data,[2,11] and since everyone in Denmark is covered by identical universal comprehensive health insurance, inferring health conditions based on ICD and ATC codes is unlikely to be influenced by the bias present in U.S. claims data, in which utilization and diagnoses are dependent on insurance coverage.

Secondary Analyses

For the three cardiovascular disease (CVD) risk factors considered as outcomes, one possible limitation of these Cox models is differential left-censoring, since the three source registers for this study—outpatient, inpatient, and prescription drug—were each established at different times. Cox model results may be biased if neighborhood deprivation is associated with the likelihood of diagnosis based on outpatient, inpatient, or prescription drug data (e.g., if there are differences in prescription or hospitalization patterns that differ by neighborhood due to practice patterns, healthcare access, or some other factor than actual disease incidence). Cox models with fixed effects also suffer from the "incidental parameters problem," and thus they were considered the secondary models in this study.[12] Logistic models were not implemented due to their inability to accommodate the very large number of variables in municipality-level fixed effects models.

Principal Component Analysis

We created a composite neighborhood deprivation index for each neighborhood by year, using principal component analysis (PCA) to combine eight neighborhood-level sociodemographic variables examined in prior observational analyses that represent different theoretical constructs capturing socioeconomic deprivation (Supplemental Table 1). We first calculated the neighborhood-level sociodemographic characteristics for each

© 2020 Hamad R et al. *JAMA Network Open.*

year and each parish. We then standardized (i.e., normalized) the neighborhood-level sociodemographic characteristics for each parish, by year. We then performed PCA, combining the neighborhood-level sociodemographic variables for each parish and year, resulting in 8 principal components for each parish and year. Consistent with prior work on neighborhood deprivation using similar techniques,[13-16] we selected the first component to serve as the composite index of neighborhood deprivation for this study. The variable loadings for this first component are shown in Supplemental Table 1. This first component explained 34% of the variance in the data (Supplemental Table 2). Similar techniques have been used to construct deprivation indices in prior studies across several international settings.[16-21]

While a composite index provides a more holistic representation of neigborhood deprivation compared with examining only a single neighborhood-level exposure (e.g., median income), it also creates difficulties in interpretation of the results. For example, different parishes may all have the same value of the composite index, and yet have very different values for the 8 different neighborhood-level characteristics. Variables loadings for each characteristics differ slightly from year to year. At the same time, a 1-unit shift in the index could result from any number of changes to the 8 different characteristics. Future work could examine each of the separate characteristics independently to tease apart their contributions to CVD risk.

In constructing the 8-variable deprivation index, we noted that several of the neighborhood characteristics were highly correlated, several had low or inconsistent variable loadings across the study period, and several variable loadings were in the opposite direction as expected (thereby complicating interpretation of the composite score). We therefore conducted a sensitivity analysis in which we narrowed the list of characteristics to those four that have been used in prior research on neighborhood deprivation in a Scandinavian context—median income, education, welfare participation, and unemployment—and whose variable loadings were in an expected direction.[13-15] In doing so, we eliminated correlated variables, those with inconsistent loadings over time, and those with low variable loadings. This is a technique that has been used in similar work in the U.S. context.[16] The first component for this 4-variable sensitivity analysis explained 56% of the variance in the data (Supplemental Table 2). We then carried out a secondary analysis in which a composite score using only these 4

© 2020 Hamad R et al. *JAMA Network Open.*

variables was the primary exposure, which resulted in findings that were similar to those of the primary analysis (Supplemental Figure 3), with increased neighborhood deprivation associated with higher risk of hypertension (0.85 percentage points per unit of deprivation index, 95%CI: 0.39, 1.30; p<0.01), hyperlipidemia (0.55 percentage points, 95%CI: 0.10, 0.99, p=0.02), diabetes (0.35 percentage points, 95%CI: 0.05, 0.65, p=0.02), and MI (0.17 percentage points, 95%CI: 0.03, 0.30, 0=0.02).

© 2020 Hamad R et al. *JAMA Network Open.*

eFigure 1. Study flowchart

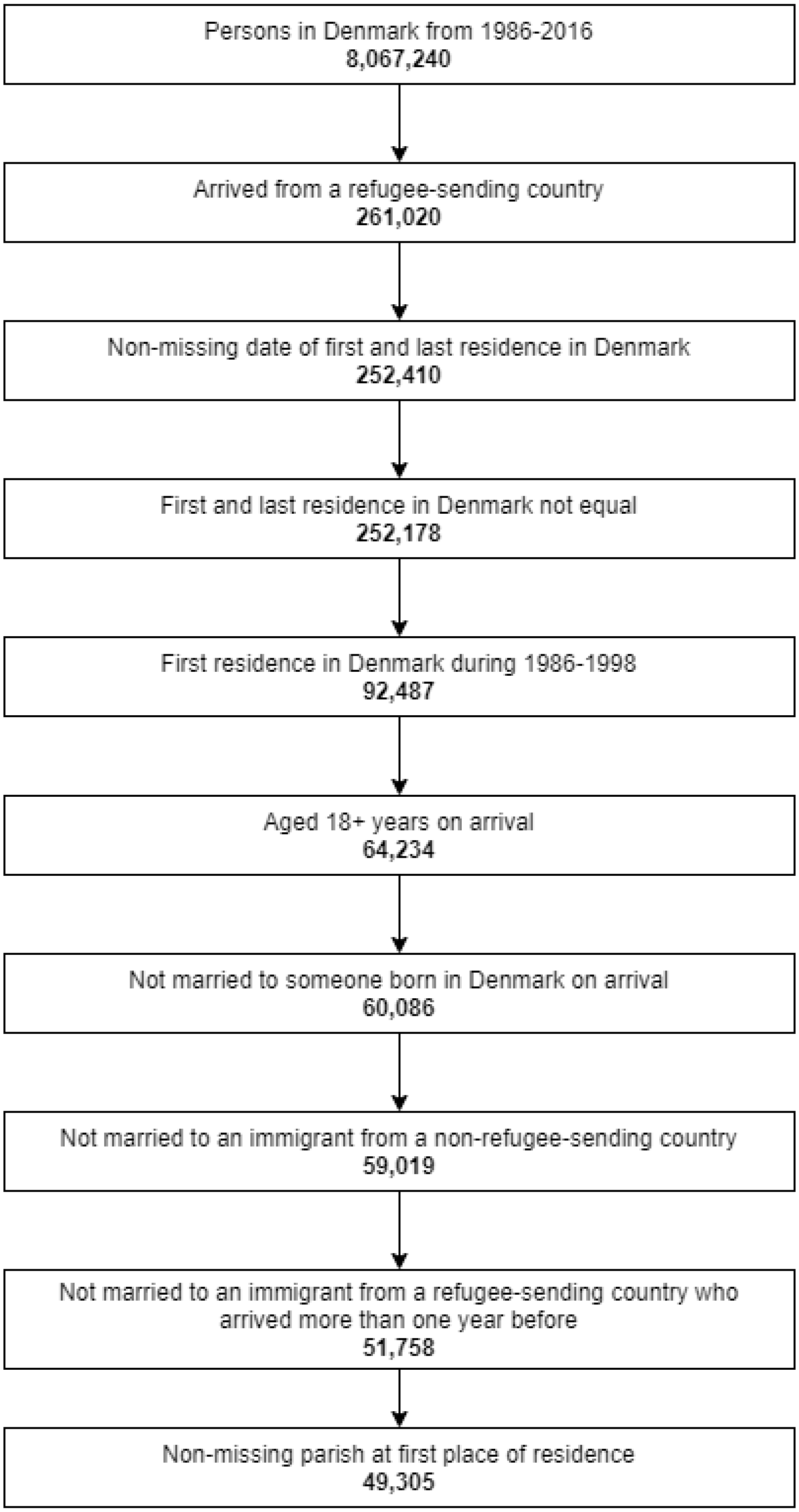

© 2020 Hamad R et al. *JAMA Network Open.*

eFigure 2. Effect of neighborhood deprivation on cardiovascular outcomes, Cox models

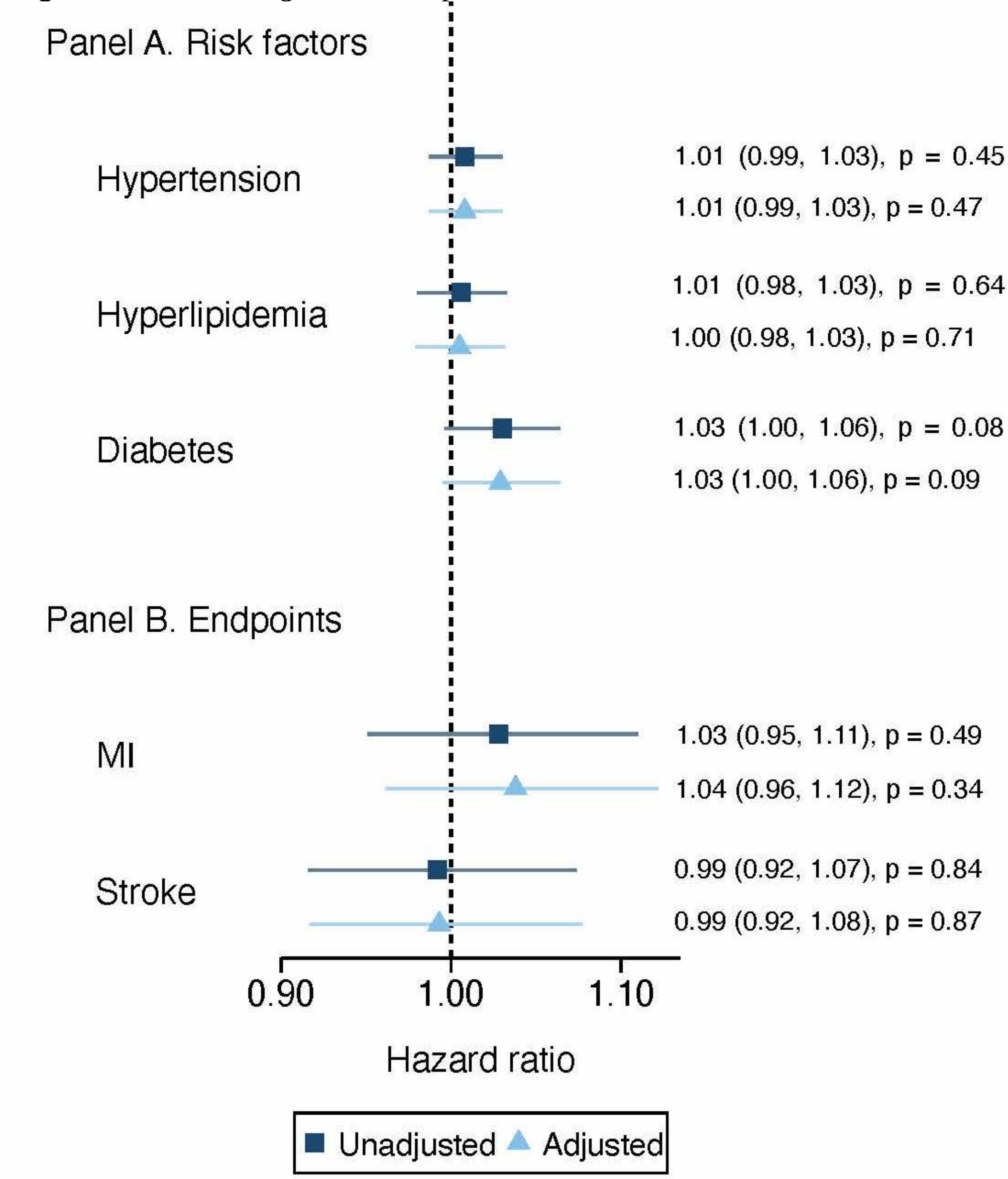

N = 49,305. Study population includes all adult immigrants (ages 18 and over), who arrived in Denmark from refugee-sending countries during 1986-1998. Diagnoses were extracted from register data using medication and physician diagnosis codes. A continuous variable representing composite deprivation index of socioeconomic deprivation was created for each neighborhood by year, using principal component analysis to combine eight neighborhood-level sociodemographic variables (Supplemental Table 1). Analyses involved multivariable Cox regressions, with covariates including family size, gender, marital status, region of origin, year of arrival, and fixed effects for municipality. Coefficients represent the change in risk (hazard ratio) per unit of the deprivation index, with 95% confidence intervals in parentheses. MI: myocardial infarction.

© 2020 Hamad R et al. *JAMA Network Open.*

eFigure 3. Effect of neighborhood deprivation on cardiovascular outcomes, using alternative neighborhood deprivation index

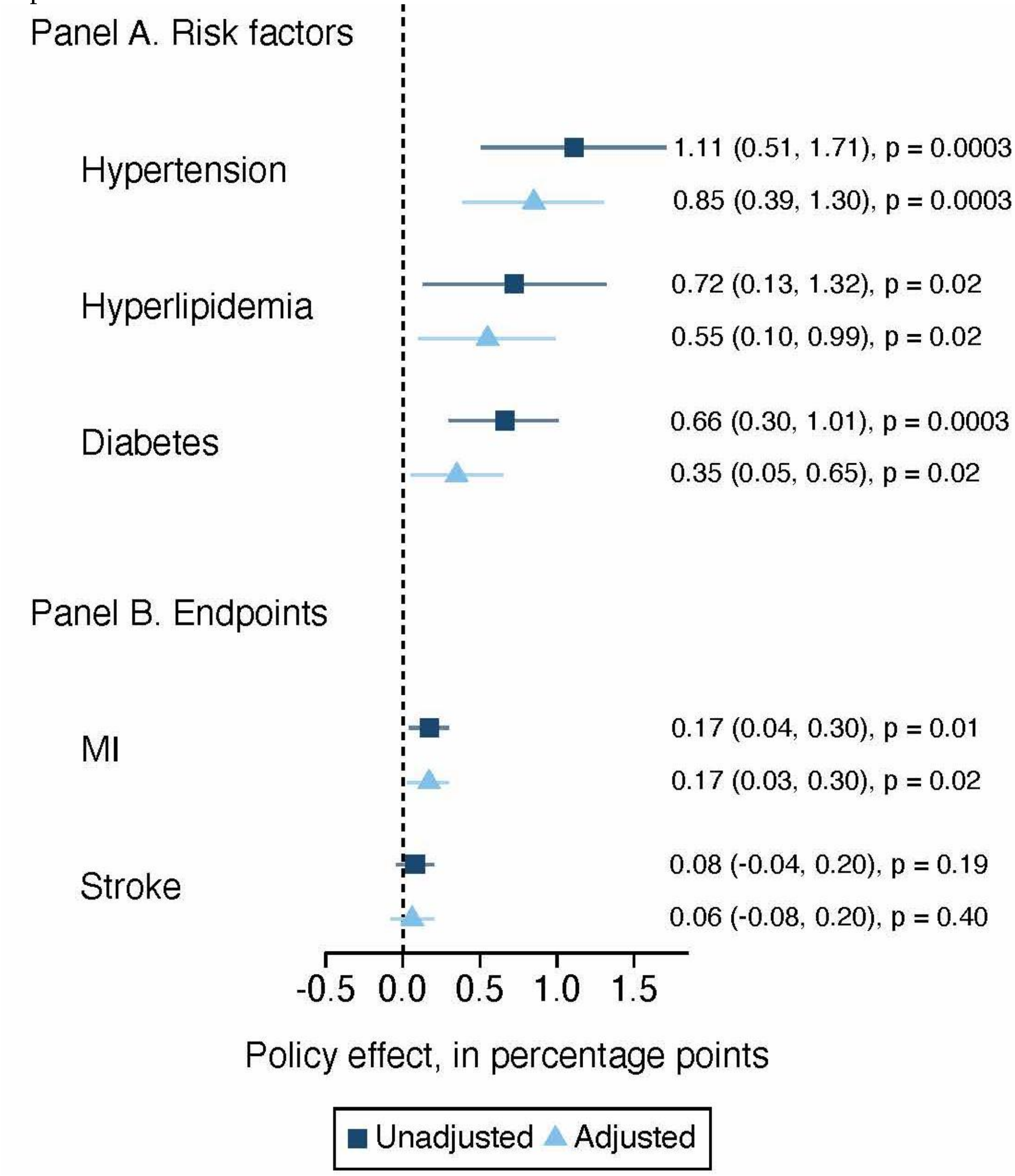

N = 49,305. Study population includes all adult immigrants (ages 18 and over) who arrived to Denmark from refugee-sending countries during 1986-1998. Diagnoses were extracted using medication and clinical encounter codes from register data. A continuous variable representing composite deprivation index of socioeconomic deprivation was created for each neighborhood by year, using principal component analysis to combine four neighborhood-level sociodemographic variables (Supplemental Table 1). Analyses involved multivariable linear regressions, with covariates including family size, gender, marital status, region of origin, year of arrival, and fixed effects for municipality. Coefficients represent the change in risk (in percentage points) per unit of the deprivation index, with 95% confidence intervals in parentheses. MI: myocardial infarction.

© 2020 Hamad R et al. *JAMA Network Open.*

**eTable 1.** Characteristics included in neighborhood deprivation index			
**Measure**	**Description**	**Variable Loading (PCA 8)**	**Variable Loading (PCA 4)**
Median income	Median family income	0.36	0.58
Education	Median years of formal schooling completed	0.43	0.51
Welfare participation	Percent receiving welfare support	-0.49	-0.48
Unemployment rate	Fraction of adults aged 18-59 who are unemployed	-0.22	-0.41
Family poverty	Percent of families in poverty	0.35	
Income inequality	Gini coefficient	0.36	
Crime rate	Violent crimes per capita	0.12	
Foreign-born	Percent of individuals who are foreign-born	0.32	

Note: Variable loadings presented above were obtained from conducting principal component analysis, averaged over the study period 1986-1998. PCA8 represents an analysis including all 8 neighborhood characteristics, while PCA4 represents an analysis conducted using 4 neighborhood characteristics that met the criteria described in the Supplemental Methods.

© 2020 Hamad R et al. *JAMA Network Open.*

eTable 2. Principal component analysis: eigenvalues and proportion of variance explained

	Component 1	Component 2	Component 3	Component 4	Component 5	Component 6	Component 7	Componnent 8
Panel A. PCA 8								
Eigenvalue	2.70	1.98	1.27	0.59	0.49	0.41	0.30	0.26
Proportion of variance explained	0.34	0.25	0.16	0.07	0.06	0.05	0.04	0.03
Panel B. PCA 4								
Eigenvalue	2.23	0.81	0.61	0.35				
Proportion of variance explained	0.56	0.20	0.15	0.09				
Note: Results presented above were obtained from conducting principal component analysis, averaged over the study period 1986-1998. PCA8 represents an analysis including all 8 neighborhood characteristics, while PCA4 represents an analysis conducted using 4 neighborhood characteristics that met the criteria described in the Supplemental Methods.								

© 2020 Hamad R et al. *JAMA Network Open.*

eTable 3. Prevalence of outcomes in refugee population compared with population of native-born Danes matched on age and gender

	Prevalence (%)	
**Health Outcome**	Refugees	Native-Born Danes
Hypertension	37.2	35.3
Hyperlipidemia	25.2	18.7
Diabetes	16.0	7.1
Myocardial infarction	3.3	2.7
Stroke	2.8	3.6

Note: N = 49,305 refugees and 493,050 native-born Danes. The comparison population of native-born Danes was created by sampling randomly from the Danish population with replacement, with 10 Danes sampled for every 1 refugee. The comparison population was matched to the refugee population by age and gender.

© 2020 Hamad R et al. *JAMA Network Open.*

eTable 4. Effect of neighborhood deprivation on cardiovascular outcomes, by gender and age of arrival in Denmark

	**Hypertension**	**Hyperlipidemia**	**Diabetes**	**MI**	**Stroke**
Panel A. By Gender					
Deprivation × Female	-0.21	-0.05	0.03	-0.12	-0.04
	(-0.68, 0.26)	(-0.48, 0.38)	(-0.44, 0.51)	(-0.33, 0.08)	(-0.20, 0.12)
Deprivation	0.80***	0.47**	0.44*	0.19***	0.02
	(0.39, 1.21)	(0.03, 0.90)	(-0.01, 0.89)	(0.05, 0.33)	(-0.15, 0.19)
Female	6.05***	-2.18***	-0.35	-2.56***	-0.51***
	(5.08, 7.01)	(-3.02, -1.34)	(-1.10, 0.40)	(-2.97, -2.14)	(-0.85, -0.18)
Panel B. By Age on Arrival					
Deprivation × Age<35	-0.09	1.16***	0.17	-0.02	-0.04
	(-0.75, 0.56)	(0.41, 1.92)	(-0.36, 0.70)	(-0.32, 0.29)	(-0.38, 0.29)
Deprivation	0.73***	0.04	0.40***	0.16**	0.05
	(0.33, 1.14)	(-0.34, 0.43)	(0.11, 0.70)	(0.03, 0.30)	(-0.07, 0.18)
Age < 35	18.82***	19.64***	11.48***	4.80***	5.23***
	(17.68, 19.95)	(18.50, 20.78)	(10.69, 12.26)	(4.28, 5.32)	(4.67, 5.78)

* p < 0.1, ** p < 0.05, *** p < 0.01

N = 49,305. Study population includes all adult immigrants (ages 18 and over) who arrived to Denmark from refugee-sending countries during 1986-1998. Diagnoses were extracted using medication and clinical encounter codes from register data. A composite deprivation index of socioeconomic deprivation was created for each neighborhood by year, using principal component analysis to combine eight neighborhood-level sociodemographic variables (Supplemental Table 1). Analyses involved multivariable linear regressions, with covariates including family size, gender, marital status, region of origin, year of arrival, and fixed effects for municipality. Coefficients represent the change in risk (in percentage points) per unit of the deprivation index, with 95% confidence intervals in parentheses. MI: myocardial infarction.

© 2020 Hamad R et al. *JAMA Network Open.*

eReferences

1. Lynge E, Sandegaard JL, Rebolj M. The Danish National Patient Register. *Scandinavian Journal of Public Health.* 2011;39(7 suppl):30-33.
2. Schmidt M, Pedersen L, Sørensen HT. The Danish Civil Registration System as a tool in epidemiology. *European journal of epidemiology.* 2014;29(8):541-549.
3. Adelborg K, Horváth-Puhó E, Ording A, Pedersen L, Toft Sørensen H, Henderson VW. Heart failure and risk of dementia: a Danish nationwide population-based cohort study. *European journal of heart failure.* 2017;19(2):253-260.
4. Corraini P, Henderson VW, Ording AG, Pedersen L, Horváth-Puhó E, Sørensen HT. Long-Term Risk of Dementia Among Survivors of Ischemic or Hemorrhagic Stroke. *Stroke.* 2017;48(1):180-186.
5. Sundbøll J, Horváth-Puhó E, Adelborg K, et al. Higher Risk of Vascular Dementia in Myocardial Infarction Survivors. *Circulation.* 2018;137(6):567-577.
6. Sundbøll J, Adelborg K, Munch T, et al. Positive predictive value of cardiovascular diagnoses in the Danish National Patient Registry: a validation study. *BMJ Open.* 2016;6(11).
7. Tessier-Sherman B, Galusha D, Taiwo OA, et al. Further validation that claims data are a useful tool for epidemiologic research on hypertension. *BMC public health.* 2013;13(1):51.
8. Poluzzi E, Strahinja P, Vargiu A, et al. Initial treatment of hypertension and adherence to therapy in general practice in Italy. *European journal of clinical pharmacology.* 2005;61(8):603-609.
9. Schmidt M, Schmidt SAJ, Sandegaard JL, Ehrenstein V, Pedersen L, Sørensen HT. The Danish National Patient Registry: a review of content, data quality, and research potential. *Clinical Epidemiology.* 2015;7:449-490.
10. Thygesen SK, Christiansen CF, Christensen S, Lash TL, Sorensen HT. The predictive value of ICD-10 diagnostic coding used to assess Charlson comorbidity index conditions in the population-based Danish National Registry of Patients. *BMC Med Res Methodol.* 2011;11:83.
11. Wallach Kildemoes H, Toft Sørensen H, Hallas J. The Danish national prescription registry. *Scandinavian journal of public health.* 2011;39(7_suppl):38-41.
12. Kalbfleisch JD, Sprott DA. Application of likelihood methods to models involving large numbers of parameters. *Journal of the Royal Statistical Society: Series B (methodological).* 1970;32(2):175-194.
13. Cubbin C, Sundquist K, Ahlén H, Johansson S-E, Winkleby MA, Sundquist J. Neighborhood deprivation and cardiovascular disease risk factors: Protective and harmful effects. *Scandinavian Journal of Social Medicine.* 2006;34(3):228-237.
14. Sundquist K, Malmstrom M, Johansson SE. Neighbourhood deprivation and incidence of coronary heart disease: a multilevel study of 2.6 million women and men in Sweden. *J Epidemiol Community Health.* 2004;58(1):71-77.
15. White JS, Hamad R, Li X, et al. Long-term effects of neighbourhood deprivation on diabetes risk: quasi-experimental evidence from a refugee dispersal policy in Sweden. *The Lancet Diabetes & Endocrinology.* 2016;4(6):517-524.
16. Messer LC, Laraia BA, Kaufman JS, et al. The development of a standardized neighborhood deprivation index. *Journal of Urban Health.* 2006;83(6):1041-1062.
17. Cubbin C, Hadden WC, Winkleby MA. Neighborhood context and cardiovascular disease risk factors: the contribution of material deprivation. *Ethn Dis.* 2001;11(4):687-700.
18. Sundquist K, Winkleby M, Ahlén H, Johansson S-E. Neighborhood socioeconomic environment and incidence of coronary heart disease: a follow-up study of 25,319 women and men in Sweden. *American journal of epidemiology.* 2004;159(7):655-662.
19. Pearce J, Blakely T, Witten K, Bartie P. Neighborhood Deprivation and Access to Fast-Food Retailing. *American Journal of Preventive Medicine.* 2007;32(5):375-382.

© 2020 Hamad R et al. *JAMA Network Open.*

20. Salmond CE, Crampton P. Development of New Zealand's deprivation index (NZDep) and its uptake as a national policy tool. *Canadian Journal of Public Health/Revue Canadienne de Sante'e Publique.* 2012;103(Supp 2):S7-S11.
21. Pampalon R, Hamel D, Gamache P, Philibert MD, Raymond G, Simpson A. An area-based material and social deprivation index for public health in Québec and Canada. *Canadian Journal of Public Health/Revue Canadienne de Sante'e Publique.* 2012;103(Supp 2):S17-S22.

© 2020 Hamad R et al. *JAMA Network Open.*
